# CO_2_ Fire Extincteurs

**Published:** 1912-12-21

**Authors:** 


### $CO_2$ Fire Extincteurs.

In our issue of November 30, 1912, dealing with modern methods of hospital construction for preventing and restricting fire, a passing reference was made to a type of extincteur with which tests and demonstration had been made in presence of our representative. This type of extincteur differentiates itself advantageously from others in that no chemical reactions have to take place inside in order to obtain the necessary propelling power. The liquid is expelled by the mechanical action of compressed carbonic-acid gas in easily applied containers. This may better be grasped by comparing it to sparklets syphons; in fact, their manufacture is carried on cheek by jowl with them in the same workshops. The extincteur acts in a similar manner, but has the much greater power necessary to project liquid some considerable distance. There are many types of extincteurs on the market, and some have already been described in our columns at length; one great objection to most is that they are charged with a solution of bicarbonate of soda and sulphuric acid. To bring such types into action a bottle containing acid is broken; the carbonic-acid gas thus formed causes an effervescing fluid of great extinguishing power to be discharged at a high velocity on to the fire. This will no doubt quench the fire, but the fluid is of such a nature that it may cause damage in the dispensary or stores.

The container of the new apparatus is made of sheet steel, coated and lined with lead, and with welded joints tested to withstand a pressure of 350 lb. to the square inch. Its capacity is about two gallons; and when charged it weighs about 30 lb., so that it can be easily handled. The "Prana" patent fire tube is made of the best brass hermetically sealed. The test witnessed by our representative consisted of lighting a wooden shed filled with paraffin-soaked shavings and resinous wood; when the flames had obtained a good hold the extincteur was addressed to the fire and completely extinguished it in a few moments. The test demonstrated that for the incipient stages of local fires the extincteur made by the British Fire Appliances Co., of Charing Cross, S.W., is reliable. Scientific tests have, however, been conducted by the British Fire Prevention Committee, and may be found in their volume No. 142. The cost of the apparatus as installed at the Royal Buckinghamshire Hospital is stated to be 35s. each, but enterprising secretaries will probably find there is some reduction where numbers are purchased.

